# Systematic analysis and identification of the absorption and metabolic components of Zengye decoction in type 2 diabetic rats by HPLC-ESI-Q-TOF–MS/MS

**DOI:** 10.1186/s13020-020-00331-z

**Published:** 2020-05-20

**Authors:** Shanquan Chang, Mei Wang, Yushan Tian, Jin Qi, Zhixia Qiu

**Affiliations:** 1 Jiangsu Key Laboratory of TCM Evaluation and Translational Research, School of Traditional Chinese Pharmacy, China Pharmaceutical University, #639 Longmian Avenue, Jiangning District, Nanjing, 211198 People’s Republic of China; 2 Department of Pharmacology of Chinese Materia Medica, School of Traditional Chinese Pharmacy, China Pharmaceutical University, #639 Longmian Avenue, Jiangning District, Nanjing, 211198 People’s Republic of China

**Keywords:** Zengye decoction, Type 2 diabetes mellitus, HPLC-ESI-Q-TOF–MS/MS, Qualitative analysis, Metabolite identification

## Abstract

**Background:**

Zengye decoction (ZYD) has been widely used in the treatment of type 2 diabetes mellitus (T2DM). Exploring the fate of various components of ZYD in vivo is of considerable significance for pharmacological research and molecular mechanism elaboration. However, the systematic analysis on the metabolic behavior of chemical components of ZYD in T2DM rats has not been reported.

**Methods:**

To screen and characterize the complex chemical compositions of ZYD, and metabolism fate in plasma, urine, bile, and feces of T2DM rats, the model of T2DM rats was prepared. A rapid procedure using high-performance liquid chromatography coupled with electrospray ionization quadrupole time of flight tandem mass spectrometry (HPLC-ESI-Q-TOF–MS/MS) was established. Data were acquired and analyzed by Agilent MassHunter Workstation Qualitative Analysis software version B.07.00 and PCDL manager B.07.00.

**Results:**

A total of 80 compounds were identified or tentatively characterized in ZYD, 31 more than previously detected. Besides, 36 prototype components and 49 metabolites of ZYD were found and characterized in T2DM rats, and the proposed fragmentation pathways and possible metabolic behaviors of the main types of compounds were described.

**Conclusions:**

This study developed the understanding of the composition of ZYD as well as the cleavage rules and metabolic pathways of the prototype compounds. Besides, this study provided abundant data for further research and for study of the metabolism of traditional Chinese medicine prescriptions.

## Background

Type 2 diabetes mellitus (T2DM) is the most common form of diabetes, accounting for 90%–95% of all diabetic patients, which is primarily due to the relative lack of insulin secretion or reduced sensitivity to insulin [1, 2]. According to the latest report, the worldwide prevalence of adult diabetes has reached 9.3%, equivalent to 463 million adults worldwide with diabetes [3]. T2DM has become a serious global public health problem. Therefore, it is of practical significance to develop new drugs for the treatment of T2DM.

Traditional Chinese medicine (TCM) has been widely used in health care in many Asian countries for thousands of years. With the release of the detailed description of TCM by the 11th version of the International Statistical Classification of Diseases and Related Health Problems (ICD), TCM is more widely and gradually accepted around the world. Zengye decoction (ZYD) is a well-known TCM prescription used to treat ‘wasting thirst syndrome’, which would probably be diagnosed as T2DM according to the nationwide unified western medicine diagnostic criteria [4, 5]. ZYD was initially recorded in *wen bing tiao bian* written by Wu tang in Qing Dynasty of Chinese history (1936 AD-1912 AD) and is composed of *Scrophulariae Radix*, *Rehmanniae Radix* and *Ophiopogonis Radix*. Modern pharmacological studies show that ZYD exhibits hypoglycemic effect [6, 7]. However, the mechanism corresponding to its hypoglycemic effect is still unclear, due to the sophisticated features of multi-components and biological multi-effect of TCM [8]. Therefore, it is necessary to evaluate the therapeutic substances of hypoglycemic effect using modern scientific research methods, not only the herbal phytochemical compositions but also the absorption and metabolism of active ingredients in vivo of T2DM.

There have been some researches on the chemical constituents and herbal ingredients of ZYD in previous studies [9, 10]. A few reports have studied the absorption of a few compounds of ZYD [11]. We have demonstrated that ZYD improves insulin resistance in T2DM rats [12]. However, the metabolism of ZYD in experimental diabetes models has not reported. Actually, in the pathological state of diabetes, the absorption, distribution, metabolism, and excretion of ZYD may be different from those in the natural and healthy state [13, 14]. This ambiguity presents the greatest obstacle to deeper pharmacological mechanism investigation and scientific connotation interpretation. Therefore, a comprehensive, systematic analysis of the absorption and metabolic components of ZYD in vivo under the diabetic state is urgent.

In this paper, a rapid procedure using high-performance liquid chromatography coupled with electrospray ionization quadrupole time of flight tandem mass spectrometry (HPLC-ESI-Q-TOF–MS/MS) was established to characterize complex chemical compounds and metabolic components. In actual, HPLC-ESI-Q-TOF–MS/MS has been substantially applied to qualitative analysis of multiple components and metabolites in the complex mixture especially for the TCM prescriptions owing to its extraordinary performance, high resolution, accurate mass measurement, and rapid scan speed [15]. In the present study, a rat model of T2DM was established, and the absorbed components and metabolic ingredients in plasma, bile, urine, feces were screened after oral administration of ZYD. At the same time, the proposed fragmentation pathways and possible metabolic behaviors of the composition of ZYD in vivo were described in detail.

## Methods

### Chemicals and reagents

HPLC-grade acetonitrile was purchased from Tedia (Fairfield, OH, USA). HPLC-grade methanol was purchased from CINC High Purity Solvents Co. Ltd (Shanghai, China). The purified water was obtained using a Milli-Q water purification system (Millipore, Bedford, MA, USA). Formic acid of HPLC-grade was purchased from Aladdin Bio-Chem Technology Co. Ltd (Shanghai, China). *n*-Butanol was purchased from Sinopharm Chemical Reagent Co. Ltd (Nanjing, China). Catalpol, leonuride, acteoside, isoacteoside, harpagide, harpagoside, were obtained from Sichuan Weikeqi biological technology Co., Ltd (purity ≥ 98%). Cinnamic acid, *p*-coumaric acid, ferulic acid, were obtained from Chengdu Biopurify Phytochemicals Ltd (purity ≥ 98%). Streptozotocin (STZ) was purchased from Sigma (St. Louis, MO, USA). All other chemicals and solvents were of analytical grade.

### Preparation of Zengye decoction extract

The crude drugs of *Scrophulariae Radix*, dried *Rehmanniae Radix*, *Ophiopogonis Radix* were purchased from Nanjing Traditional Chinese Medicine Clinics (Nanjing, China). The three herbs were authenticated by professor Jin Qi (Jiangsu Key Laboratory of TCM Evaluation and Translational Research). The crude drugs (130 g. *Scrophulariae Radix*: dried *Rehmanniae Radix*: *Ophiopogonis Radix*, 5:4:4, w/w/w) were soaked in double-distilled water and extracted three times by boiling in distilled water (1300 mL, 1040 mL, 780 mL) under reflux for 1 h. And then, the collected filtrates were combined, concentrated, and freeze-dried to obtain a lyophilized powder.

### Preparation model of T2DM rats and drug administration

Male Wistar rats weighing 140–160 g were purchased from Comparative Medicine Center of Yangzhou University (Yangzhou, China). The rats were raised in an air-conditioned room at 23 ± 1 °C and a 12 h light/dark cycle. The animals were adaptively fed for 1 week prior to use. All the operations were allowed by the Animal Ethics Committee of China Pharmaceutical University, China Pharmaceutical University, Nanjing, Jiangsu, China.

The model of T2DM rats was induced by high-fat diet combined with low-dose STZ (35 mg/kg) [16]. The method was improved according to the previous reports and laboratory studies [10, 17]. Briefly, the animals were fed a high-fat diet for 3 weeks followed by intraperitoneal injection of STZ (35 mg/kg) which dissolved in cold citrate buffer (pH 4.3, 0.05 M). The fasting blood glucose (FBG) of rats was measured 3 days after injection, the level was higher than 11.1 mmol/L for subsequent experiments.

The T2DM rats were randomly divided into two groups. One group received ZYD (13 g/kg body weight, twice a day) via oral administration for 7 days. The other group received water, as the model control group. In addition, a normal healthy rats group received water, as the normal control group. The rats were fasted but with free access to water for 12 h before experiment.

### Samples collection and pretreatment

All samples were obtained after drug administration. The blood samples (n = 4) were collected from T2DM rats in the heparinized centrifugal tube at 0.5 h, 1 h, 2 h, 4 h and 8 h by retro-orbital venipuncture and immediately centrifuged at 1200×*g* for 15 min to obtain plasma. The urine and feces (n = 4) were collected at 0–24 h in independent metabolic cages. The feces samples were naturally dried in the fume hood and then crushed into powder. The bile (n = 4) was collected at 0–8 h by bile duct intubation and drainage under general anesthesia induced by 1% pentobarbital sodium, 55 mg/kg. All biological samples of the same type in the same group at each time point were equally combined into one sample, and stored at − 80 °C before pretreatment and analysis.

An aliquot of 1 mL plasma sample was added into triple volume of acetonitrile and vigorously vortexed for 1 min. Then the mixture was centrifuged at 1500 xg for 15 min. The supernatant was transferred to another centrifuge tube and evaporated to dryness under a gentle stream of nitrogen at 37 °C. The residual was reconstituted in 200 μL methanol: water mixture (7:3, v/v) and then centrifuged at 13,700×*g* for 15 min. The supernatant was filtered through 0.22 μm nylon microporous filter membrane. The filtrates were analyzed by HPLC-ESI-Q-TOF–MS/MS.

Likewise, an aliquot of 1 mL urine sample was added into 3 mL methanol and vortexed for 1 min. A weight of 0.8 g feces was extracted within 8 mL methanol for 30 min under ultrasonic. Afterwards, the mixture was centrifuged at 13,700×*g* for 15 min. The supernatant was transferred to another centrifuge tube and evaporated to dryness under a gentle stream of nitrogen at 37 °C. The residual was re-dissolved in 200 μL reconstituted solvent (methanol: water, 7:3, v/v) and centrifuged at 13,700×*g* for 15 min, and the solution was filtered through 0.22 μm nylon microporous filter membrane. The bile was pretreatment in the same way as urine.

### HPLC-ESI-Q-TOF–MS/MS condition

Chromatographic separation was performed on an Agilent 1260 Infinity HPLC system (Agilent Technologies, Santa Clara, CA, USA) using a Diamonsil C18 column (4.6 × 250 mm, 5 μm) and a precolumn with the same packing at 30 °C. The mobile phase A was water containing 0.01% formic acid and phase B was acetonitrile containing 0.01% formic acid. The gradient elution conditions were as follows: 2% B at 0–10 min; 2–7% B at 10–20 min; 7–16% B at 20–45 min; 16–22% B at 45–60 min; 22–34% B at 60–85 min; 34–80% B at 85–115 min; 80–100% B at 115–125 min. The total flow rate was 1 mL/min, and an aliquot of 20 μL injected for analysis.

Mass spectrometry data were obtained using an Agilent 6530 Q-TOF MS/MS system (Agilent Technologies, Santa Clara, CA, USA) equipped with an ESI interface. The optimum parameters were set as follows: ESI source in the negative mode; drying gas (N_2_) flow rate 10.0 L/min; drying gas temperature 320 °C; nebulizer pressure 35 psi; capillary voltage 3500 V; skimmer 65 V; fragmentor voltage 120 V; MS/MS collision energy 25 V; scan range 100–2000 Da.

The HPLC-ESI-Q-TOF–MS/MS data were acquired and analyzed by an Agilent MassHunter Workstation Qualitative Analysis software version B.07.00 and PCDL manager B.07.00 (Agilent Technologies, Santa Clara, CA, USA).

## Results

### Identification of chemical profile of Zengye Decoction

**Fig. 1:**
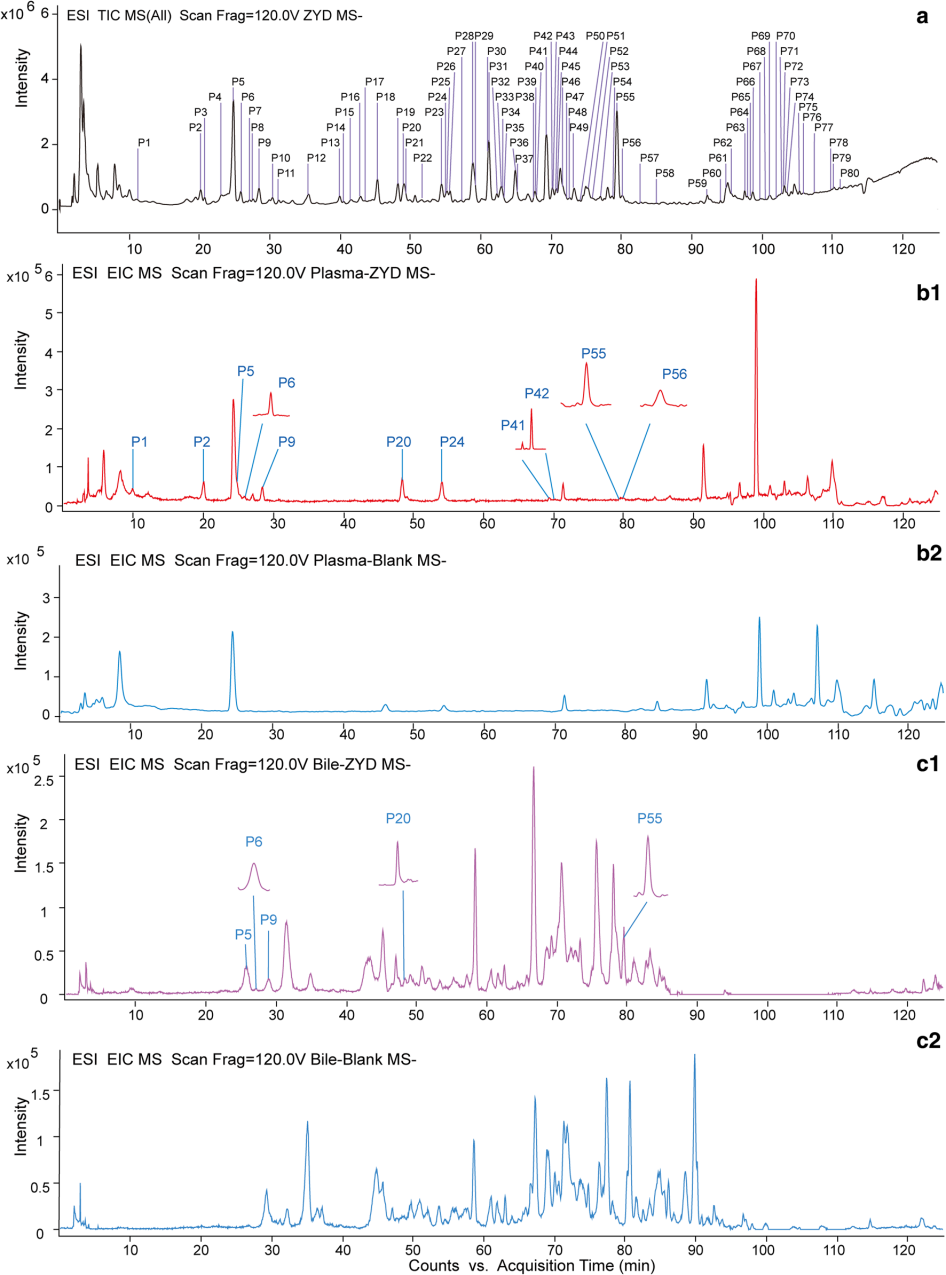

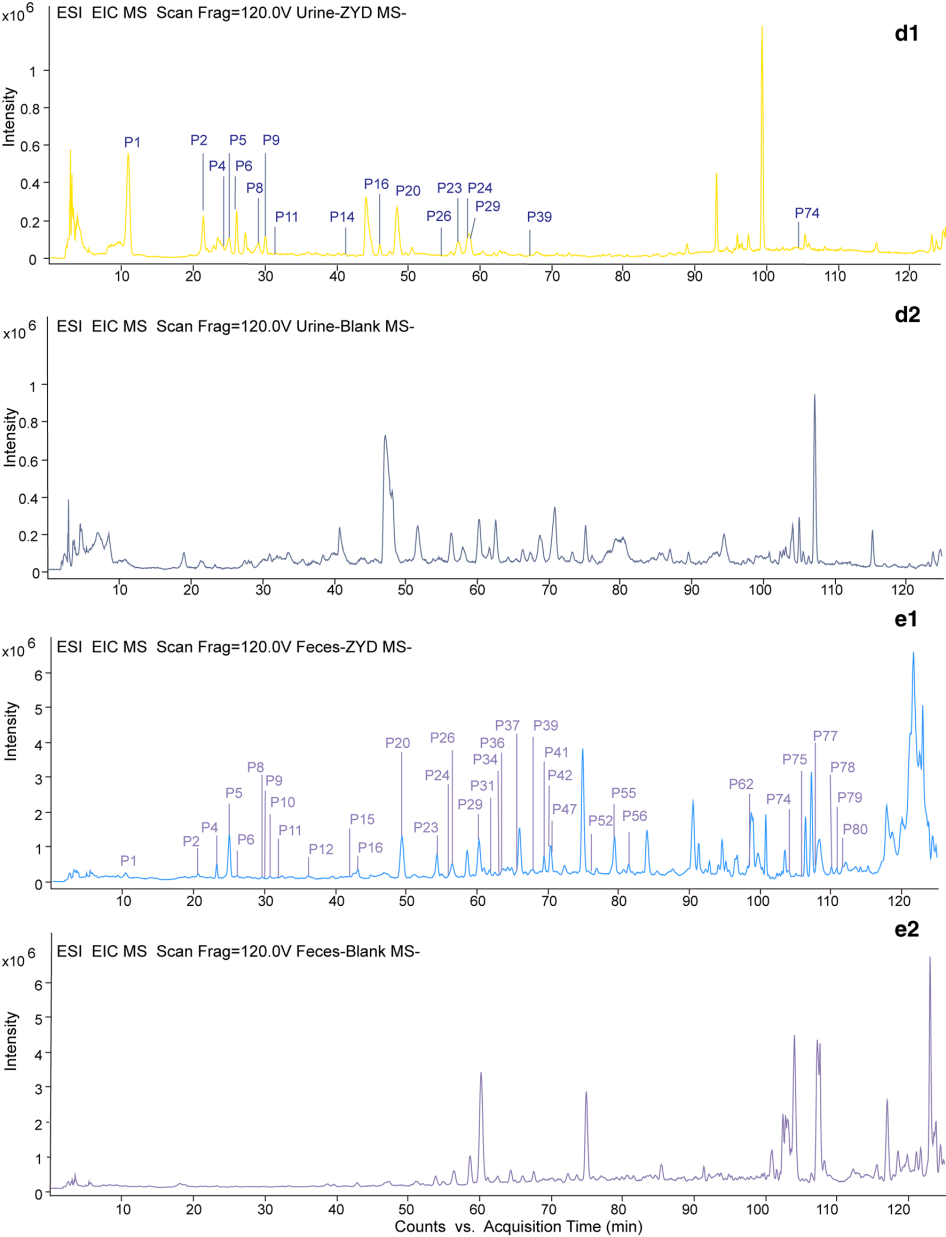
**a** Total ion chromatogram (TIC) of Zengye decoction (ZYD) in the negative ion mode and **b**–**e** extracted ion chromatograms (EICs) of ZYD in biological and blank samples in the negative ion mode. **b**-1 ZYD plasma sample and **b**-2 blank plasma sample; **c**-1 ZYD bile sample and **c**-2 blank bile sample; **d**-1 ZYD urine sample and **d**-2 blank urine sample; and **e**-1 ZYD feces sample and **e**-2 blank feces sample

In order to more accurately characterize the intracorporal process of ZYD in the T2DM rats, the chemical constituents of ZYD were initially identified by HPLC-ESI-Q-TOF–MS/MS. In the present study, 80 compounds from ZYD were tentatively identified by comparing with the reference standards, the retention time, and reviewing literature [9, 10, 19–25]. The total ion chromatogram (TIC) of ZYD in negative mode was showed in Fig. 1a, and the detailed compounds information was summarized in Additional file 1: Table S1. Thirty-one previously undetected compounds were found compared with previous reports [9, 10]. In addition, the structural types of all compounds in ZYD were mainly iridoid glycosides, phenethylalchohol glycosides and phenylpropanoid glycosides, aromatic acid, homoisoflavonoids, steroidal saponins.

### Characterization of ZYD prototype components absorbed in T2DM rats

**Fig. 2:**
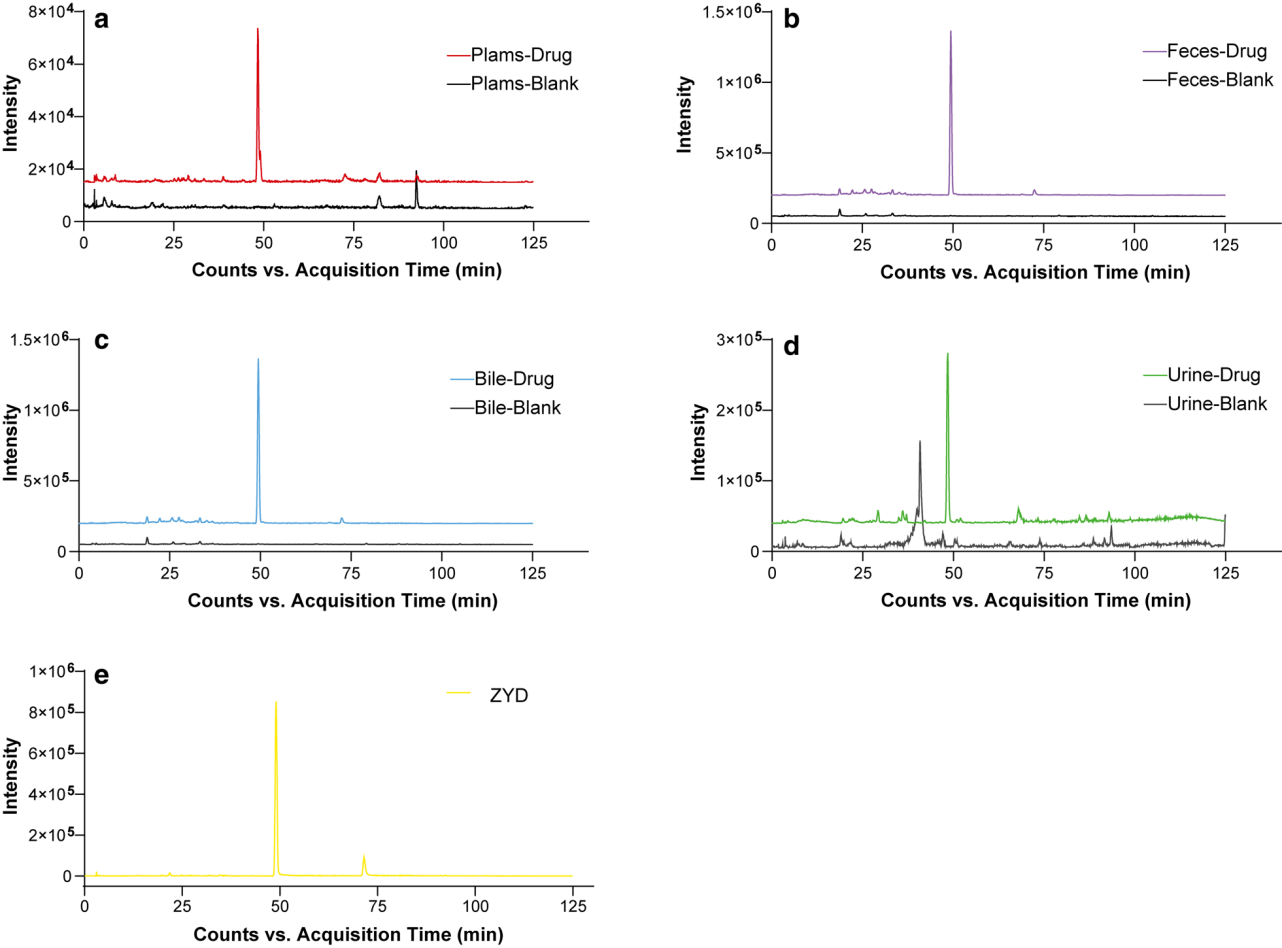
EICs of rehmapicrogenin at m/z 183.1027, [M−H]^−^ in negative ion mode; **a** ZYD-containing and blank plasma sample; **b** ZYD-containing and blank feces sample; **c** ZYD-containing and blank bile sample; **d** ZYD-containing and blank urine sample; **e** ZYD

In this study, the biological samples, including plasma, urine, feces, and bile of T2DM rats treated with ZYD, were analyzed by HPLC-ESI-Q-TOF–MS/MS under constant conditions. Peaks which displayed at the same position on the chromatograms of both drug-containing biological samples and ZYD, but not in controlled blank biological samples were considered as absorbed and metabolic components of ZYD. Both TIC and EIC (extracted ion chromatograms) profiles were used to screen the absorbed prototype components. Finally, 11 compounds were observed in plasma, 17 were in urine, 5 were in bile, 35 were in feces in T2DM rats treated with ZYD. The EICs were showed in Fig. 1b1–e2 and the prototype compounds were listed in Table 1. Furthermore, rehmapicrogenin was chosen as a representative absorbed component to demonstrate EICs further in Fig. 2. In contrast to the controlled blank biological samples, rehmapicrogenin showed remarkable peaks in drug-containing groups.

**Table 1:** Characterization of ZYD prototype components in vivo by HPLC-ESI-Q-TOF-MS/MS

Peak No.	t_R_(min)	Precursor ions (*m/z*)		Formula	Error (mDa)	Fragment ions	Identification	P	U	F	B	Refs.
		Experimental	Theoretical									
P1	10.332	407.1282*	407.1195	C_17_H_22_O_10_	− 8.70	361.1038, 199.0607, 181.0507, 169.0505	Catalpol	+	+	+	−	[10]
P2	20.658	391.1284*	391.1246	C_15_H_22_O_19_	− 3.81	211.1011, 183.0662, 165.0543, 139.0410	Aucubin	+	+	+	−	[10]
P4	23.285	731.2325*	731.2251	C_27_H_42_O_20_	− 7.35	685.2276, 505.1599, 341.1118, 179.0561	Rehmannioside D	−	+	+	−	[10]
P5	25.065	409.1384*	409.1351	C_15_H_24_O_10_	− 3.05	201.0771, 183.0663, 165.0556, 157.0504	Harpagide	+	+	+	+	[10]
P6	26.149	393.1443*	393.1402	C_15_H_24_O_9_	− 4.06	185.0560, 179.0526, 167.0716, 113.0238	Leonuride	+	+	+	+	[10]
P8	29.733	373.1216	373.1140	C_16_H_22_O_10_	− 7.58	331.0989, 221.0893, 167.0426, 149.0622	Geniposidic acid	−	+	+	−	[18]
P9	30.113	421.1332*	421.1351	C_16_H_24_O_10_	1.95	213.0732, 195.0662, 183.0657, 169.0437	6-*O*-methylcatalpol	+	+	+	+	[19]
P10	30.783	461.1698	461.1664	C_20_H_30_O_12_	− 3.35	461.1698	Decaffeoylacteoside	−	−	+	−	[10]
P11	31.790	375.1317	375.1297	C_16_H_24_O_10_	2.03	375.1317	8-epilogonic acid	−	+	+	−	[9]
P12	36.172	487.1527	487.1457	C_21_H_28_O_13_	− 6.99	487.1527	Cistanoside F	−	−	+	−	[10]
P14	41.765	313.0938	313.0929	C_14_H_18_O_8_	− 0.91	313.0938, 229.1749, 137.0605, 123.0439	Rhamnopyranosyl vanilloyl	−	+	−	−	[20]
P15	42.066	475.1845	475.1821	C_21_H_32_O_12_	− 2.4	475.1845	Darendoside B	−	−	+	−	[10]
P16	43.203	607.2290	607.2244	C_26_H_40_O_16_	− 4.64	607.2298, 475.1859, 461.1697, 443.1586, 149.0457, 131.0351	β-(3-hydroxy-4-methoxyhenyl) ethyl-*O*-α-L-arabinopyranosyl-(1 → 6)-*O*-[6-α-L-rhamnpyranosyl-(1 → 3)-β-D-glucopyranoside	−	+	+	−	[10]
P20	49.444	183.1028	183.1027	C_10_H_16_O_3_	− 0.13	183.1036, 139.1123, 123.0792	Rehmapicrogenin	+	+	+	+	[10]
P23	54.309	435.2269*	435.2236	C_19_H_34_O_8_	− 3.33	389.2370, 179.0539, 161.0451, 119.0348	Rehmaionoside A/B	−	+	+	−	[10]
P26	56.018	799.2778	799.2666	C_36_H_48_O_20_	− 11.18	799.2778	Jionoside A1/A2	−	+	+	−	[21]
P24	56.295	163.0399	163.0401	C_9_H_8_O_3_	0.17	145.0270, 119.0499	*p*-coumaric acid	+	+	+	−	[10]
P29	60.152	193.0511	193.0500	C_10_H_10_O_4_	− 0.47	178.0269, 149.0598, 134.0369, 121,0282	Ferulic acid	−	+	+	−	[10]
P31	61.894	623.2049	623.1981	C_29_H_36_O_15_	− 6.76	461.1676, 315.1084, 161.0241, 135.0445	Acteoside	−	−	+	−	[10]
P34	63.221	769.2634	769.2561	C_35_H_46_O_19_	− 7.35	769.2634	Scrophuloside B1/B2	−	−	+	−	[22]
P36	63.473	623.2021	623.1981	C_29_H_36_O_15_	− 3.96	623.2021	Isoacteoside or Forsythoside A	−	−	+	−	[10]
P37	65.538	525.1665	525.1614	C_24_H_30_O_13_	− 5.14	525.1665	8-*O*-caffeoyl harpagide	−	−	+	−	[19]
P39	67.938	429.2171	429.2130	C_21_H_34_O_9_	− 4.09	249.1512, 231.1370, 187.1472	Jiocarotenoside A1/A2	−	+	+	−	[23]
P41	69.475	783.2798	783.2717	C_36_H_48_O_19_	− 8.10	607.2294, 589.2154, 461.1666, 193.0500	Angoroside C	+	−	+	−	[10]
P42	70.273	783.2840	783.2717	C_36_H_48_O_19_	− 12.30	783.2840, 829.4135	Isoangoroside C	+	−	+	−	[10]
P47	70.428	637.2199	637.2138	C_30_H_38_O_15_	− 6.11	461.1706, 193.0512, 149.0577, 134.0372	Leucosceptoside A	−	−	+	−	[21]
P52	75.561	651.2397	651.2294	C_31_H_40_O_15_	− 10.26	651.2397	Cistanoside D	−	−	+	−	[24]
P55	79.343	539.1823*	539.1770	C_24_H_30_O_11_	− 5.29	493.1715, 345.1210, 183.0662, 165.0556	Harpagoside	+	−	+	+	[10]
P56	81.455	147.0450	147.0452	C_9_H_8_O_2_	0.15	147.0450	Cinnamic acid	+	−	+	−	[10]
P62	98.429	345.1014	345.0980	C_18_H_18_O_7_	− 3.42	345.1014	5,7,2′,4′-tetradihydroxy-8-methoyl-6-methyl-homoisoflavanone	−	−	+	−	[10]
P74	104.020	359.1161	359.1136	C_19_H_20_O_7_	− 2.47	359.1161	Ophiopogonanone E	−	+	+	−	[10]
P75	105.884	343.1215	343.1187	C_19_H_20_O_6_	− 2.79	343.1215	5-7-4′-trihydroxy-5′-methoxy-6,8-dimethyl hamoisoflavanone	−	−	+	−	[25]
P77	107.848	327.0905	327.0874	C_18_H_16_O_6_	− 3.09	327.0905	Ophiopogonone A	−	−	+	−	[10]
P78	110.065	339.0898	339.0874	C_19_H_16_O_6_	− 2.39	339.0898	Methylophiopogone A	−	+	+	−	[10]
P79	110.853	341.1059	341.1031	C_19_H_18_O_6_	− 2.84	206.0587, 178.0258	Methylophiopogonanone A	−	−	+	−	[10]
P80	111.626	327.1260	327.1238	C_19_H_20_O_5_	− 2.20	327.1260	Methylophiopogone B	−	−	+	−	[10]

a), t_R_, retention time; *, [M+HCOOH−H]^−^, other, [M−H]^−^; P, plasma; U, urine; F, feces; B, bile. +, containing; −, not

### Identification of phenethylalchohol glycosides and phenylpropanoid glycosides

**Fig. 3:**
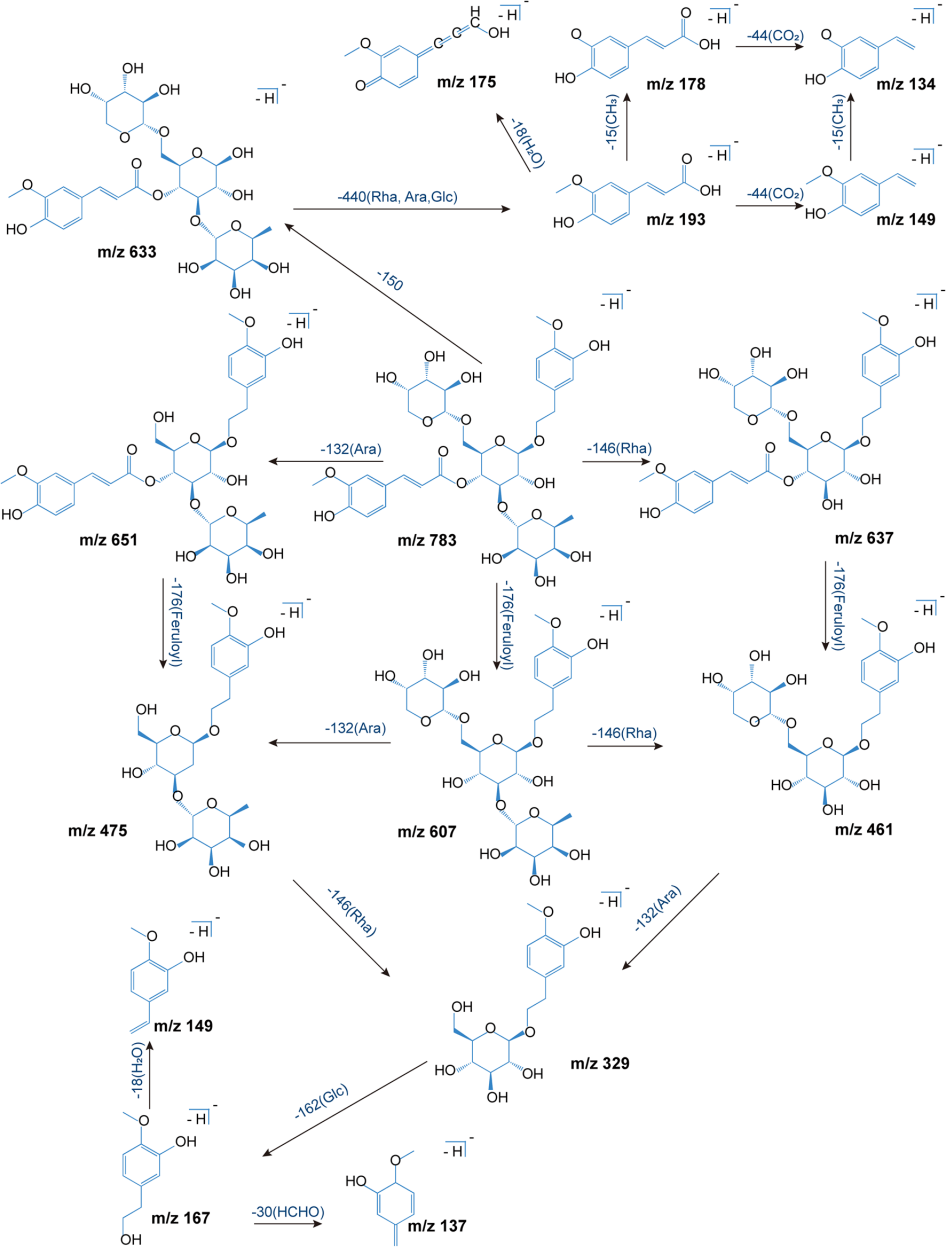
Proposed fragmentation pathway diagram of angoroside C in the negative ion mode. Ara, arabinosyl; Rha, rhamnosyl; Glc, glucosyl; Feruloyl, Ferulic acid dehydration

Totally 11 phenethylalchohol glycosides and phenylpropanoid glycosides were detected in vivo, and their structures usually included phenylethanol, phenylpropanoid, and glycoside units. Phenylethanol group often involved loss of neutral molecules like H_2_O, HCHO, and CH_3_OH. Phenylpropionic acid easily loses H_2_O due to the presence of hydroxyl and carboxyl groups. In another way, different phenylpropionic acid shows different typical fragments, such as ferulic acid (193, 178, 175, 149, 134), *p*-coumaric acid (163, 145, 119), cinnamic acid (147, 129, 103). In the case of angoroside C, the fragmentation pathway diagram was shown in Fig. 3. The quasi-molecular ion peak of angoroside C (783.2798 [M−H]^−^) could lose a series of residuals to generate ion peaks including 651.2173 [M-Ara(132 Da)–H]^−^, 633.2078 [M-Ara-H_2_O–H]^−^, *m/z* 607.2299 [M-Feruloyl(176 Da)-H]^−^, *m/z* 589.2192 [M-Feruloyl-H_2_O–H]^−^, *m/z* 637.2163 [M-Rha(146 Da)–H]^−^, *m/z* 475.1792 [M-Ara-Feruloyl-H]^–^, *m/z* 461.1707 [M-Rha-Feruloyl-H]^−^, *m/z* 443.1553 [M-Rha-Feruloyl-H_2_O–H]^–^, *m/z* 329.1226 [M-Ara-Rha-Feruloyl-H]^−^, *m/z* 311.1474 [M-Rha-Ara-H_2_O–H]^–^, and typical fragments where the residues further to break, such as feruloyl (175,134), 3-methylhydroxytyrosol (167), etc. Likewise, other compounds could produce similar fragments.

### Identification of iridoid glycosides

**Fig. 4:**
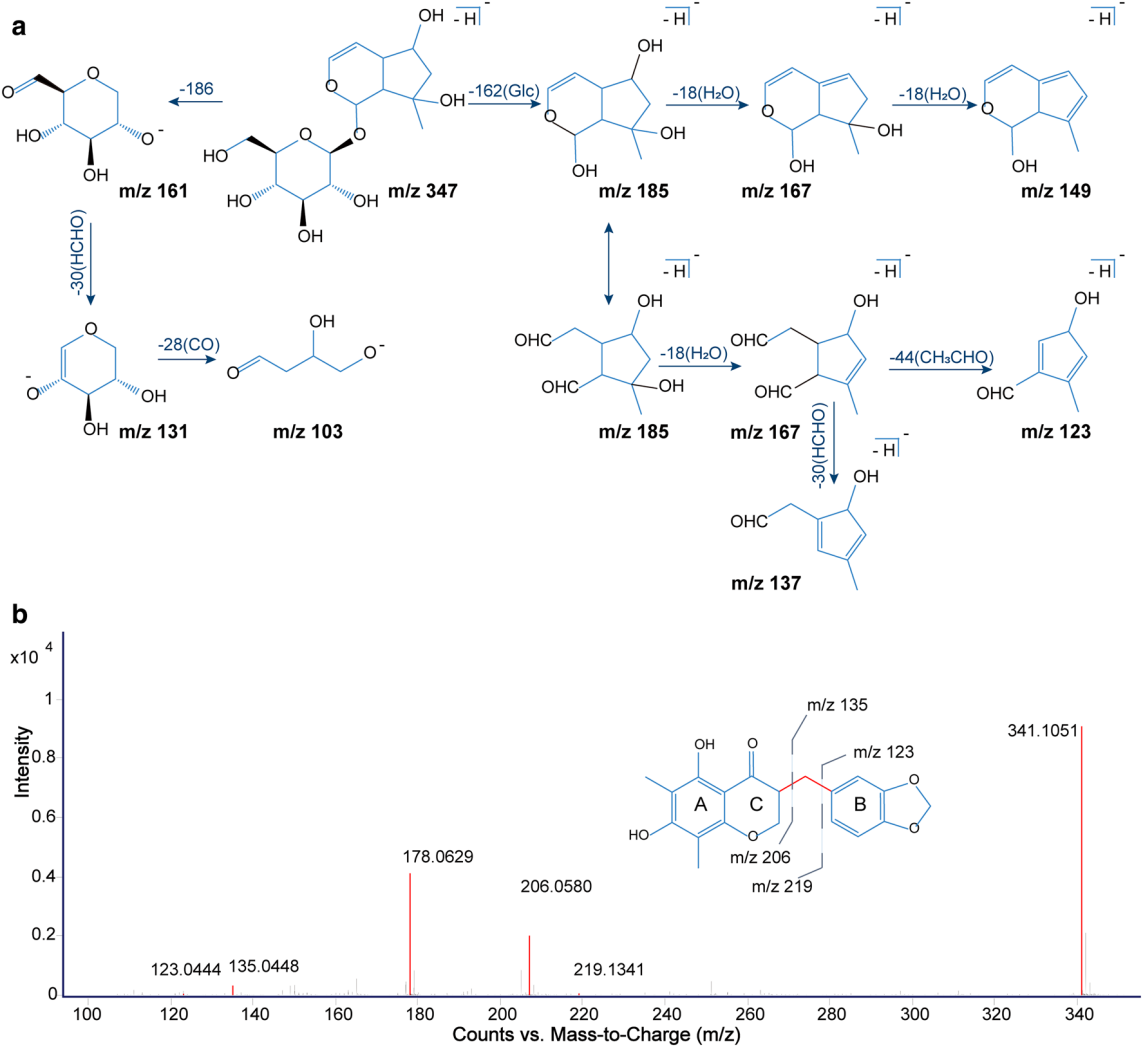
Proposed fragmentation pathways of **a** leonuride and **b** methylophiopogonanone A in the negative ion mode

Iridoid glycosides are the main components of *Scrophulariae Radix* and dried *Rehmanniae Radix*, which have similar mother nuclei and mass spectral fragmentation pattern during cracking. These compounds generally produce precursor ions like [M−H]^−^, [M+HCOOH–H]^−^, [M+Cl]^−^, and [2M−H]^−^ in the negative ion mode. In the secondary ion mode, the glucosyl group (Glc, C_6_H_10_O_5_, 162 Da) were usually preferentially lost to expose iridoid mother nucleus. Subsequent loss of a series of H_2_O (18 Da) molecules occurred because of the presence of many hydroxyl groups (OH, 17 Da) in the mother nucleus. Fracture of the iridoid at enol-ether bonds was accompanied by the loss of acetaldehyde (CH_3_CHO, 44 Da), formaldehyde (HCHO, 30 Da), and H_2_O. Typical fragmentation pathway provided reliable information for the identification of iridoid glycosides. For instance, leonuride was the main iridoid glycosides in *Rehmanniae Radix*. The precursor ions were *m/z* 393.1503 [M+HCOOH–H]^−^, *m/z* 383.1210 [M+Cl]^−^, *m/z* 347.1439 [M−H]^−^, which produced *m/z* 185.0860 [M-Glc-H]^−^, *m/z* 167.0741 [M-Glc-H_2_O–H]^−^, *m/z* 149.0606 [M-Glc-2H_2_O–H]^−^, *m/z* 137.0203 [M-Glc-HCHO–H]^−^, *m/z* 123.0444 [M-Glc-CH_3_CHO–H]^−^. At the same time, the glucosyl residue also produced typical fragments such as *m/z* 161.0462, *m/z* 113.0227. In order to more intuitively display the fragmentation pathway of this type components, the proposed fragmentation pathways of leonuride in negative ion mode was exhibited in Fig. 4a.

### Identification of homoisoflavones

Seven homoisoflavones were detected in T2DM rats. Homoisoflavones are a particular class of flavonoids, which are connected by methylene group (CH_2_) between B and C rings. Compound P79 was confirmed as methyl-ophiopogonanone A, the MS/MS spectrum and proposed fragmentation diagram were shown in Fig. 4b. P79 gave [M−H]^−^ at *m/z* 341.1051, in which fragmentation at *m/z* 206.0580 that originated from the initial loss of B-ring and CH_2_ to generate [M-*B*-*ring*-CH_2_–H]^−^. The ion at *m/z* 178.0629 was attributed to a loss of CO from *m/z* 206.0580. Besides, [M−H]^−^ ion just eliminated B-ring to produce the [M-*B*-*ring*-H]^−^ at *m/z* 219.1341. Analogously, the remaining six compounds were identified, respectively.

### Identification of other compounds

Based on their exact molecular mass and MS/MS spectra, P14, P15, P20, P23, and P39 were temporarily identified as rhamnopyranosyl vanilloyl, darendoside B, rehmapicrogenin, rehmaionoside A/B, jiocarotenoside A1/A2, respectively.

### Tentative characterization of the ZYD metabolites in T2DM rats

Potential metabolic pathways of the ZYD components were determined by comparing data from public databases and relevant publications with the cleavage results of ZYD component mother nuclei. As a result, a total of 49 presumptive metabolites in plasma, feces, urine, and bile were preliminary illuminated. All metabolic components were listed in Table 2.

**Table 2:** Identification of ZYD metabolic components in T2DM rats

Peak No.	t_R_(min)	Precursor ions (m/z)		Error (mDa)	Formula	Fragment ions(m/z)	Identification	Metabolic type	P	U	F	B
		Exp	The									
M1	12.985	201.0776	201.0768	− 0.75	C_9_H_14_O_5_	201.0762, 157.0529, 139.0751	Harpagenin	Hydrolyzation	+	−	**+**	−
M2	24.773	377.1460	377.1453	− 0.68	C_16_H_26_O_10_	377.1460, 217.0938, 169.0841, 161.0465	Dihydrogen methylcatalpol	Methylation, hydrogenation	+	**+**	−	−
M3	26.057	263.0240	263.0231	− 0.90	C_9_H_12_O_7_S	263.0240, 183.0455, 165.0588, 121.0291	Dehydrated harpagenin sulfate	Dehydration, sulfation	−	+	−	−
M4	26.304	153.0555	153.0557	0.22	C_8_H_10_O_3_	153.0576, 123.0448, 135.0412, 121.0281	Hydroxytyrosol	Hydrolyzation	+	−	+	−
M5	26.862	583.1938	583.2032	9.43	C_27_H_36_O_14_	583.2279, 195.0671, 151.0671, 179.0566, 161.0460, 149.0501	Acetyl 6-O- dihydro-feruloyl harpagide	Hydrogenation, acetylation	−	−	+	−
M6	29.227	185.0819	185.0819	0.13	C_9_H_14_O_4_	185.0819, 141.0891	Deglucosylated leonuride	Deglucosylation	+	+	−	+
M7	36.029	225.0774	225.0768	− 0.55	C_11_H_14_O_5_	225.0801, 210.0793, 165.0556	Methyl hydrated ferulic acid	Methylation, hydration	−	−	+	−
M8	31.271	181.0506	181.0506	0.03	C_9_H_10_O_4_	181.0513, 163.0398, 137.0514, 119.0498	Hydrated *p*-coumaric acid	Hydration	+	+	+	+
M9	35.171	451.2234*	451.2185	− 4.91	C_19_H_34_O_9_	451.2234, 225.0505, 179.0553, 161.0482	Hydroxy rehmaionoside A	Hydroxylation	−	−	+	−
M10	36.81	363.1109	363.1085	− 2.36	C_18_H_20_O_8_	363.1136, 345.1053, 183.0626, 179.0445, 165.0555, 139.0667	Deglucosylated 8-O-caffeoyl-harpagide	Deglucosylation	−	−	+	−
M11	37.114	137.0602	137.0608	0.6	C_8_H_10_O_2_	137.0602, 122.0397, 111.0424, 107.0455	Deoxyhydroxytyrosol	Deoxidation	−	−	+	−
M12	37.366	489.1641	489.1614	− 2.74	C_21_H_30_O_13_	489.1641, 179.0363, 165.0542, 113.0249	Didehydrated 8-O-caffeoyl harpagide	Dehydration	−	−	−	+
M13	37.895	339.0709	339.0722	1.26	C_15_H_16_O_9_	339.0709	*p*-coumaric acid glucuronide	Glucuronidation	−	−	−	+
M14	37.895	181.0506	181.0506	0.03	C_9_H_10_O_4_	181.0506, 163.0773, 137.0601,	Dihydro-caffeic acid	Hydrogenation	−	+	+	−
M15	39.276	151.0405	151.0405	− 0.43	C_8_H_8_O_3_	151.0405, 123.0439, 107.0501	Dehydrogen hydroxytyrosol	Dehydrogenation	−	+	+	−
M16	39.401	371.0993	371.0984	− 0.93	C_16_H_20_O_10_	371.0993, 195.0657, 177.0549, 193.0334, 175.0255, 113.0242	Dihydro-ferulic acid glucuronide	Hydrogenation, glucuronidation	+	+	−	−
M17	41.295	369.0842	369.0827	− 1.48	C_16_H_18_O_10_	369.0842, 193.0504, 178.0272, 149.0599, 134.0369, 113.0232	Ferulic acid glucuronide	Glucuronidation	+	+	−	+
M18	41.618	233.0131	233.0125	− 0.57	C_8_H_10_O_6_S	233.0131, 153.0554, 135.0444, 123.0449, 121.0299, 109.0274	Hydroxytyrosol sulfate	Sulfation	−	+	+	−
M19	42.554	341.0890	341.0878	− 1.19	C_15_H_18_O_9_	341.0890, 165.0556, 121.0657, 175.0233, 149.0598, 113.0236	Dihydro-*p*-coumaric acid glucuronide	Hydrogenation, glucuronidation	−	+	−	−
M20	42.703	357.0818	357.0827	0.92	C_15_H_18_O_10_	357.0822, 339.0646, 175.0585, 131.0406	Hydrated *p*-coumaric acid glucuronide	Glucuronidation, hydration	−	−	+	−
M21	45.675	181.0870	181.0870	0.02	C_10_H_14_O_3_	181.0376, 137.0971, 122.0663, 121.0653	Dehydro-rehmapicrogenin	Dehydrogenation	+	−	+	−
M22	45.826	179.0350	179.0350	− 0.02	C_9_H_8_O_4_	179.0613, 135.0450, 161.4731,	Caffeic acid	Hydrolyzation	−	+	+	−
M23	47.591	359.1361	359.1348	− 1.34	C_16_H_24_O_9_	359.1361, 183.1025, 139.1122, 113.0232	Rehmapicrogenin glucuronide	Glucuronidation	−	+	−	+
M24	47.813	151.0398	151.0401	0.27	C_8_H_8_O_3_	151.0398, 107.0500	Dehydrogen hydroxytyrosol	Dehydrogenation	−	+	−	−
M25	48.821	247.0288	247.0282	− 0.62	C_9_H_12_O_6_S	247.0288, 167.0711, 152.0474, 149.0285	Methyl hydroxytyrosol sulfate	Methylation, sulfation	−	+	−	−
M26	49.757	385.1154	385.1140	− 1.38	C_17_H_22_O_10_	385.1154, 209.0821, 191.0689, 175.0120, 113.0244	Dihydro-methyl ferulic acid glucuronide	Hydrogenation, methylation, glucuronidation	−	+	−	+
M27	50.395	369.0827	369.0827	0.02	C_16_H_18_O_10_	369.0842, 193.0504, 178.0272, 149.0599, 134.0369, 113.0232	Ferulic acid glucuronide	Glucuronidation	−	−	−	+
M28	54.181	275.0239	275.0231	− 0.8	C_10_H_12_O_7_S	275.0239, 195.0662, 177.0558, 151.0761, 136.0524, 121.0293	Dihydro-ferulic acid sulfate	Hydrogenation, sulfation	−	+	−	−
M29	54.078	195.0667	195.0663	− 0.42	C_10_H_12_O_4_	195.0676, 177.0570, 136.0524	Dihydro-ferulic acid	Hydrogenation	+	+	+	−
M30	55.035	165.0556	165.0557	0.12	C_9_H_10_O_3_	165.0559, 147.0427, 129.0326, 121.0655	Hydrated cinnamic acid	Hydration	−	−	+	+
M31	55.744	233.0139	233.0125	− 1.37	C_8_H_10_O_6_S	233.0131, 153.0554, 135.0444, 123.0449	Hydroxytyrosol sulfate	Sulfation	−	−	+	−
M32	55.195	331.1208	331.1187	− 2.09	C_18_H_20_O_6_	331.1222, 313.1108, 287.0830, 165.0553, 147.0440, 103.0543	Deglucosylated harpagoside	Deglucosylation	−	−	+	−
M33	56.101	521.1820	521.1664	− 12.05	C_25_H_30_O_12_	521.1785, 503.1661, 183.0665, 157.0509, 193.0404, 113.0247	Dehydrated harpa-gide glucuronide	Dehydration, glucuronidation	−	−	+	−
M34	56.427	245.0133	245.0125	− 0.77	C_9_H_10_O_6_S	245.0133, 165.0554, 147.0432, 121.0657	Dihydro-*p*-coumaric acid sulfate	Hydrogenation, sulfation	−	+	−	−
M35	58.309	209.0822	209.0819	− 0.27	C_11_H_14_O_4_	209.0813, 191.0713, 165.0926, 149.0616	Dihydro-methyl ferulic acid	Hydrogenation, methylation	+	−	+	−
M36	58.362	273.0083	273.0074	− 0.85	C_10_H_10_O_7_S	273.0083, 193.0503, 178.0268, 134.0369	Ferulic acid sulfate	Sulfation	−	+	−	−
M37	59.756	625.2194	625.2194	− 5.61	C_29_H_38_O_15_	625.2194, 461.1695, 315.1104, 181.0510, 163.0407, 153.0538	Dihydro-acteoside	Hydrogenation	−	−	+	−
M38	60.258	217.1088	217.1081	− 0.65	C_10_H_18_O_5_	217.1087, 199.0936, 186.2198, 171.1025, 155.1062, 153.0895	Dihydro-methyl harpagenin	Hydrogenation, methylation	−	−	+	−
M39	61.965	135.0449	135.0452	0.25	C_8_H_8_O_2_	135.0449, 123.0065, 107.0468, 100.9257	Dehydrated hydroxytyrosol	Dehydration	+	−	+	−
M40	63.176	361.1518	361.1504	− 1.39	C_16_H_26_O_9_	361.2317, 185.1180, 141.1279, 113.0242	Dihydrogen rehmapicrogenin glucuronide	Hydrogenation, glucuronidation	−	+	−	−
M41	64.035	247.0294	247.0282	− 1.22	C_9_H_12_O_6_S	247.0537, 167.0706, 152.0476	Methyl hydroxytyrosol sulfate	Methylation, sulfation	−	−	+	−
M42	66.026	377.1471	377.1453	− 1.78	C_16_H_26_O_10_	377.1471, 201.1129, 183.1002, 165.0567, 175.0242, 113.0242	Harpagenin glucuronide	Glucuronidation	−	+	−	+
M43	70.428	637.2199	637.2138	− 6.11	C_30_H_38_O_15_	637.2155, 461.1706, 193.0512, 135.0407	Methyl acteoside	Methylation	−	−	+	−
M44	72.673	275.0246	275.0231	− 1.50	C_10_H_12_O_7_S	275.0240, 195.0665, 177.0575, 151.0772	Hydrated ferulic acid sulfate	Hydration, sulfation	+	−	+	−
M45	72.935	245.0135	245.0125	− 0.97	C_9_H_10_O_6_S	245.0135, 165.0666, 147.0549, 121.0355	Dihydro-*p*-coumaric acid sulfate	Hydrogenation, sulfation	−	−	+	−
M46	78.821	273.0081	273.0074	− 0.65	C_10_H_10_O_7_S	273.0090, 193.0506, 178.0271, 134.0372	Ferulic acid sulfate	Sulfation	+	−	−	+
M47	78.166	149.0609	149.0608	− 0.10	C_9_H_10_O_2_	149.0609, 107.0480, 105.0703	Dihydro-cinnamic acid	Hydrogenation	−	−	+	−
M48	79.350	583.2092	583.2032	− 5.97	C_27_H_36_O_14_	583.2100, 193.0525, 149.0439, 201.1145, 183.0656, 165.0568	Acetyl-6-O-dihydro-feruloyl harpagide	Hydrogenation, acetylation	−	−	+	−
M49	74.738	315.1270	315.1297	2.67	C_14_H_20_O_8_	315.1278, 297.1136, 161.0582, 135.0442	Hydroxytyrosol glucosylate	Hydrolyzation	−	−	+	−

a): P, plasma; U, urine; F, feces; B, bile. +, containing; −, not

### Identification of iridoid glycoside-related metabolites

**Fig. 5:**
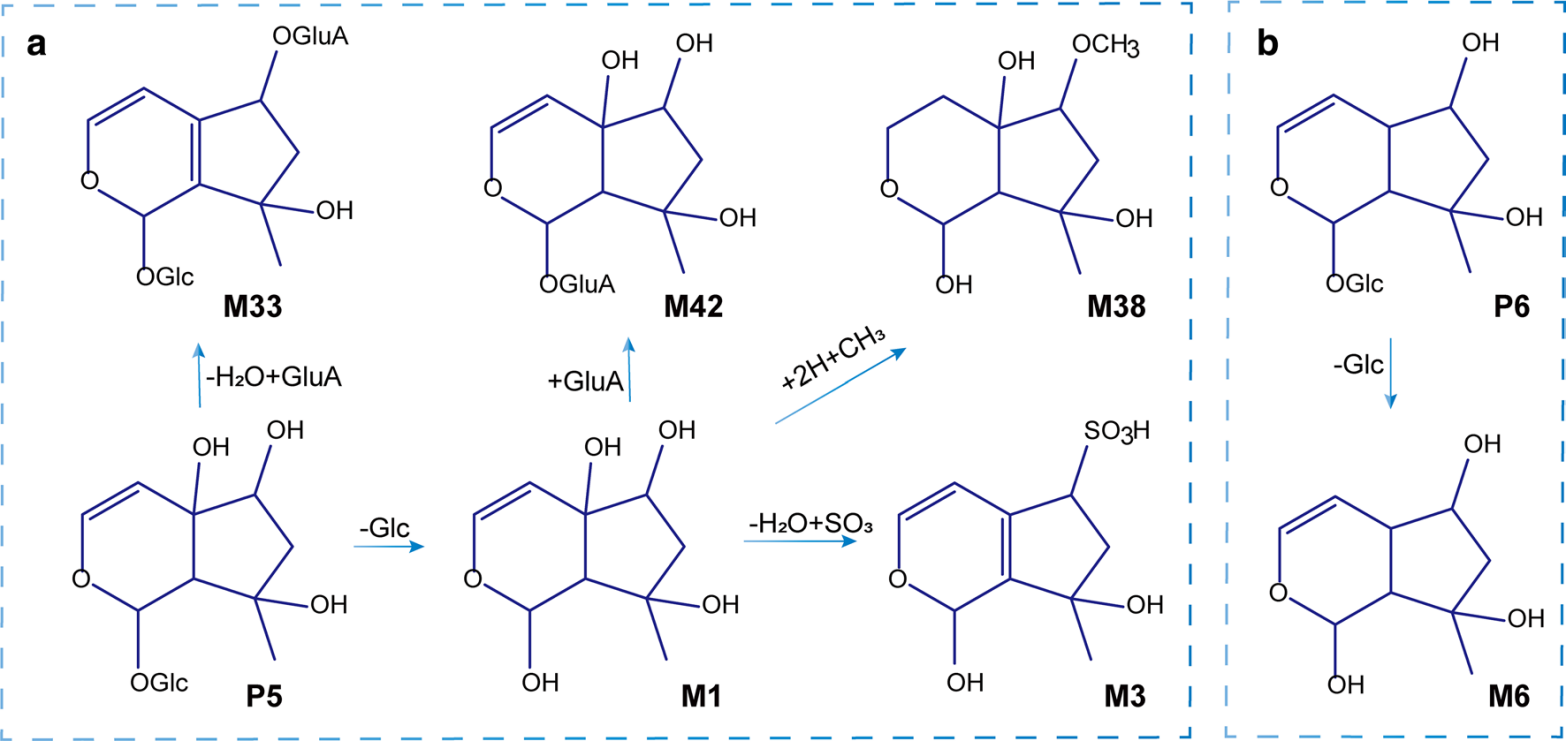
Possible metabolic pathways of major iridoid glycosides in T2DM rats. **a** Harpagide; **b** Leonuride

Totally 12 metabolites of the iridoids were initially identified, most of which were derived from the metabolism of harpagide and its derivatives. Harpagide and its derivatives easily lose a glycosyl group and are converted to harpagenin by hydrolases. M1 displayed [M−H]^−^ at *m/z* 201.1129, which typical product ions were basically consistent with the above description of harpagide. M42 showed [M−H]^−^ at *m/z* 377.1471, and the fragment ion at *m/z* 201.1129 was yielded obviously by the loss of a GluA group (176 Da). Simultaneously, the deglucose products of some other iridoid glycosides were also found. M6 displayed [M−H]^−^ at *m/z* 185.0819, a neutral loss of 162 Da (Glc) comparing with leonuride, indicating it was deglucosylated leonuride. M32 exhibited [M−H]^−^ at *m/z* 331.1208. Comparing to harpagoside, the metabolite underwent a loss of glucosyl group. Possible metabolic pathways of harpagide and leonuride were shown in Fig. 5a, b.

### Identification of phenylpropanoid-related metabolites

**Fig. 6:**
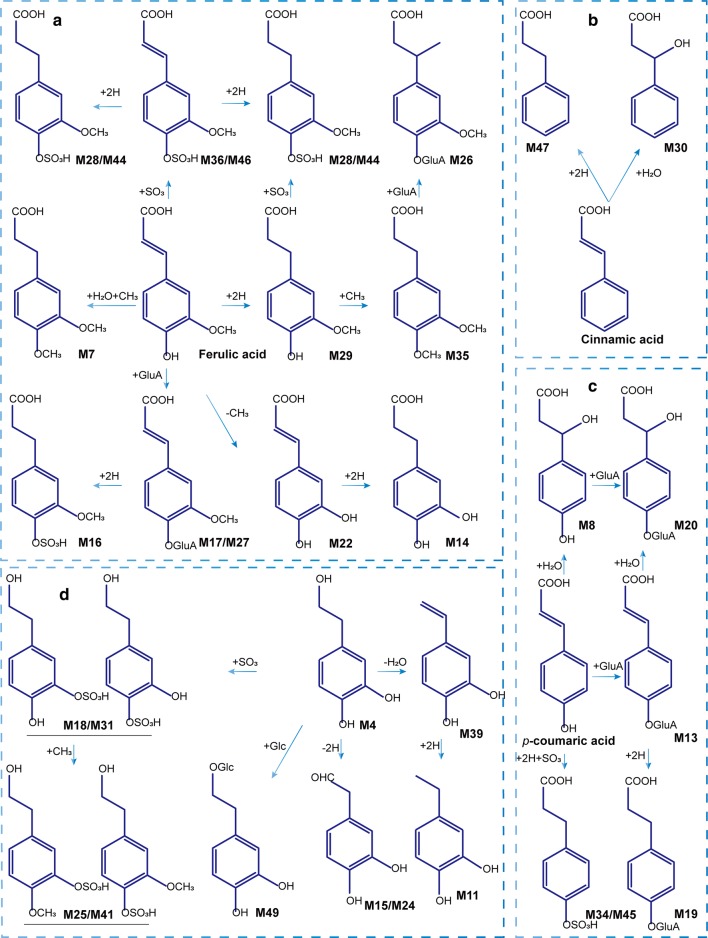
Possible metabolic pathways for major ZYD compounds in T2DM rats. **a** ferulic acid; **b** cinnamic acid; **c***p*-coumaric acid; and **d** Hydroxytyrosol

A total of 23 constituents were initially identified as generating from the metabolism of phenylpropanoid-related compounds. The metabolic pathways were shown in Fig. 6a–c. [M−H]^–^ ions, M19 at *m/z* 341.0890 and M34 at *m/z* 245.0133, had formed by the addition of 176 Da (GluA, C_6_H_8_O_7_) and 80 Da (SO_3_), respectively, to *m/z* 165.0554. Both had neutral losses of H_2_O and CO_2_ (44 Da). The metabolic profile was consistent with *p*-coumaric acid, indicating that the two metabolites were the glucuronidation and sulfonation products of dihydro *p*-coumaric acid. At the same time, the demethylation and hydrogenation products of ferulic acid were also observed. The demethylated product M22, called caffeic acid, neutral losing CO_2_ to produce abundant fragments of *m/z* 135.0450. The neutral loss of hydrogenated product M29 was the same as that of ferulic acid, including the loss of H_2_O to produce *m/z* 177.0570, and the loss of CO_2_ and methyl group (CH_3_, 15 Da) to produce typical debris *m/z* 136.0524, etc. The other 19 metabolites were identified in a similar manner.

### Identification of hydroxytyrosol-related metabolites

Hydroxytyrosol (HT) was mainly derived from phenylethanoid glycosides, which was produced by hydrolysis and formed different metabolites through various metabolic pathways in vivo. In this study, ten HT-related metabolites were identified in vivo, and the specific metabolic pathways were shown in Fig. 6d. M15 showed [M−H]^–^ at *m/z* 151.0405, the loss of 2 Da (2H) from HT, suggesting that the metabolite was generated from the dehydrogenation of HT. Moreover, M18 and M31 both yielded [M−H]^−^ at *m/z* 233.0139, linkage of an SO_3_ group separately, suggesting it to be hydroxytyrosol sulfated. Furthermore, both M25 and M41 showed [M−H]^−^ at *m/z* 247.0288, the addition of methyl to M18 and M31, respectively.

### Identification of metabolic components of other compounds

Analysis of the fragmentation pattern of rehmaionoside A found that M9 showed [M+HCOOH-H]^–^ at *m/z* 451.2234, an addition of 16 Da to [rehmaionoside A-H]^−^, suggesting that M9 was tentatively identified as hydroxyrehmaionoside A. The product ion *m/z* 243.1105, and 225.0782 were losses of glucosyl group and water from M9. The product ions of M23 included *m/z* 183.1025, *m/z* 139.1122, *m/z* 175.0294, and *m/z* 113.0232. *m/z* 183.1025, and *m/z* 139.1122 were product ions of P20 and *m/z* 175.0294, *m/z* 113.0232 were the characteristic fragments of glucuronic acid. M23 was thus initially identified as a glucuronic acid-binding product of rehmapicrogenin. Also, the dehydrogenation product M21 (*m/z* 181.087) of rehmapicrogenin, and the hydrogenation product M40 (*m/z* 361.1518) of M23 were observed. The above fragmentation regularity was consistent with rehmapicrogenin.

### Distribution of ZYD metabolites in T2DM rats

According to the distribution of ZYD metabolites in T2DM rats, Fourteen metabolites were found in plasma samples, 23 in urine, 33 in feces, and 11 in bile, which all have been identified and the details were listed in Table 2. Harpagoside, an iridoid glycoside, was the primary bioactive constituent of ZYD, which was detected in plasma, bile and feces samples of T2DM rats and its possible metabolites could be found in all bio-samples. For example, deglucosylation of harpagoside was observed in feces due to the transformation of harpagoside by glycoside hydrolase in the gut. Besides, glucuronic conjugates of *p*-coumaric acid, harpagenin, ferulic acid, and rehmapicrogenin were detected in bile, which was consistent with the glucuronic conjugates as the primarily metabolites in bile excretion. Meanwhile, *p*-coumaric acid was found in plasma, urine, and feces, but not in bile. It was likely that *p*-coumaric acid was metabolized to a glucuronic conjugate in the liver and then excreted into the duodenum via the bile duct and regenerated the prototype by a glucuronidase, and some of them were reabsorbed into the liver through the enterohepatic circulation.

## Discussion

According to the results of the pre-experiment, there was no significant difference between the previously established method and the current method in the HPLC optimization process [10]. Hence, the previous performance was used for the next experiment. Besides, the negative ion mode was chosen for further analysis because most of the compounds in ZYD contain functional groups such as hydroxyl, carboxyl, etc., and most components can be detected in the negative ion mode.

Sufficient detection level was the premise of instrument analysis [26]. The low concentrations of TCM components in vivo and possibly substantial interference by the matrix effect posed significant challenges for the analysis in vivo [27]. Samples handling has become the essential part of biological sample analysis, so it was of considerable significance to choose the appropriate pretreatment method. In the current research, dialysis, protein precipitation (PPT), solid phase extraction (SPE), immunoaffinity extraction, and other methods were widely used for the biological samples pretreatment. Among them, the PPT shows the advantages of simplicity, rapidity, and convenience, and it has been widely used in the qualitative analysis of TCM in vivo [28]. So PPT was the preferred method for this experiment.

The effectiveness of TCM for disease prevention and treatment depends on the active ingredients contained in TCM [29]. The absorption of a drug is a prerequisite for its pharmacological activity within the body. Therefore, it was assumed that the absorbable component might be an active ingredient, and that the disease condition might have some impacts on the absorption process [30]. Diabetes may reduce the expression and function of P-glycoprotein (P-gp) in the intestine [31]. When the intestinal P-gp activity is inhibited, the absorption of some drugs will be enhanced in the intestine [32]. A previous study found that the plasma concentrations of catalpol and harpagide in diabetes-model rats were increased compared with normal rats, and that the clearance rate was slower [11]. Diabetes also changes the experesion of cytochrome P450 enzymes, including CYP3A4, CYP2E1, CYP2C9, and CYP2D4 participate in phase I drug metabolism [33–35]. Catalpol has been shown to effect the activity of CYP3A4, CYP2E1 and CYP2C9 that resulted in pharmacokinetic interactions of coadministered drugs [36]. It is possible that differences in the pharmacokinetics of ZYD compounds in diabetic and normal rats may be caused by disease-associated changes in some functional enzymes.

Some of the prototype and metabolic components identified in ZYD have had pharmacological effects in the treatment of T2DM in animal models and in cell lines. Harpagoside in ZYD could activate the PPAR-γ pathway in 3T3-L1 adipocytes to regulate lipid and glucose metabolism similar to the hypoglycemic effects of thiazolidinedione [37, 38]. *p*-Coumaric acid was shown to promote glucose uptake and utilization by activating the AMPK pathway and upregulating GLUT2 expression [39, 40]. Ferulic acid was reported to promote glucose uptake by activating PI3K-Akt pathway and upregulating the expression of GLUT4. It has also been found to promote glycogen synthesis and inhibit gluconeogenesis by downregulating the expression of PEPCK, G6PC and upregulating glucokinase expression [41–44]. In addition, Ferulic acid was also found to increase intracellular Ca^2+^ to promote insulin secretion [44, 45]. Among metabolites, caffeic acid was shown to increase insulin sensitivity in HepG2 cells, reduce hepatic glucose output, enhance glucose uptake, promote insulin secretion, and increase antioxidant activity in adipocytes [45–48]. Our previous study showed that ZYD had hypoglycemic activity, improved dyslipidemia, and promoted pancreatic islet-cell function in T2DM model rats [12]. Knowing which of the chemical constituents of ZYD presented in vivo is essential for further investigation of the material basis and mechanism of ZYD in the treatment of T2DM.

## Conclusions

ZYD is increasingly used for the treatment of T2DM. However there have been few reports on the component analysis of ZYD in vivo and even fewer in the pathological state of T2DM. This study identified and tentatively characterized previously undetected ZYD ingredients and was the first to systematically analyze the metabolism of ZYD ingredients in T2DM rats. As a consequence, thirty-six prototype components and 49 metabolites were presumed and characterized in vivo, and the proposed fragmentation pathways and possible metabolic behaviors of the main types of compounds were analyzed. In summary, this study added to the understanding of the chemical profile of ZYD and its metabolism information in T2DM rats. It provided essential data for the detailed pharmacokinetic study and pharmacodynamic material basis of ZYD in T2DM. Meanwhile, the study methods are applicable to the study of the metabolism of other TCM prescriptions.

## Supplementary information

**Additional file 1: Table S1.** Compounds information of ZYD by HPLC-ESI-Q-TOF MS/MS.

## Data Availability

The dataset supporting the conclusions of this article is included within the article.

## Abbreviations

Ara: Arabinosyl group
FBG: Fasting blood glucose
Glc: Glucosyl group
GluA: Glucuronic acid group
HT: Hydroxytyrosol
PPT: Protein precipitation
Rha: Rhamnosyl group
T2DM: Type 2 diabetes mellitus
TCM: Traditional Chinese medicine
STZ: Streptozotocin
ZYD: Zengye decoction
