# Supplementary material for: Systematic analysis and identification of the absorption and metabolic components of Zengye decoction in type 2 diabetic rats by HPLC-ESI-Q-TOF–MS/MS
Source: Chin Med. 2020 May 20;15:50. doi: 10.1186/s13020-020-00331-z (PMC7238542; doi:10.1186/s13020-020-00331-z)
Supplement: Supplementary file 1 — Additional file 1: Table S1. Compounds information of ZYD by HPLC-ESI-Q-TOF MS/MS. [file 13020_2020_331_MOESM1_ESM.docx]

**Additional material**

Table S1 Compounds information of ZYD by HPLC-ESI-Q-TOF MS/MS

| No | t_R_(min) | Formula | *m/z* | Fragment ions | Compounds | Origins |
| --- | --- | --- | --- | --- | --- | --- |
| P1 | 10.012 | C_15_H_22_O_10_ | 407.1305* | 407.1194 [M+HCOOH-H]^-^, 199.0622 [M-H-Glc]^-^, 169.0497 [M-H-Glc-HCHO]^-^, 181.0489 [M-H-Glc-H_2_O]^-^ | Catalpol ^a^ | RR |
| P2 | 20.188 | C_15_H_22_O_9_ | 391.1338* | 391.1244 [M+HCOOH-H]^-^, 183.0656 [M-H-Glc]^-^, 165.0564 [M-H-Glc-H_2_O]^-^ | Aucubin | SR |
| P3 | 20.717 | C_21_H_32_O_14_ | 553.1928* | 345.1136 [M-H-Glc]^-^, 179.0550 [Glu-H]^-^, 165.0553 [M-H-Glc-H_2_O]^-^, 147.0447 [M-H-Glc-2H_2_O]^-^ | 6-*O*-β-glucosylaucubin^#^ | SR |
| P4 | 23.034 | C_27_H_42_O_10_ | 731.2467* | 731.2469[M+HCOOH-H]^-^, 685.2408[M-H]^-^, 721.2185[M+Cl]^-^ | Rehmannioside D | RR |
| P5 | 24.797 | C_15_H_24_O_10_ | 409.1454* | 409.1343 [M+HCOOH-H]^-^, 201.0755 [M-H-Glc]^-^, 183.0665 [M-H-Glc-H_2_O]^-^, 165.0557 [M-H-Glc-2H_2_O]^-^ | Harpagide ^a^ | SR |
| P6 | 25.855 | C_15_H_24_O_9_ | 393.1503* | 393.1414 [M+HCOOH-H]^-^, 185.0810 [M-H-Glc]^-^, 167.0717 [M-H-Glc-H_2_O]^-^, 149.0608 [M-H-Glc-2H_2_O]^-^ | Leonuride ^a^ | RR |
| P7 | 27.013 | C_21_H_32_O_15_ | 571.2045* | 571.2029 [M+HCOOH-H]^-^, 525.1977[M-H]^-^, 561.1753[M+Cl]^-^ | Stachysoside A | SR |
| P8 | 27.568 | C_16_H_22_O_10_ | 373.1233 | 211.0608 [M-H-Glc]^-^, 167.0715 [M-H-Glc-CO_2_]^-^, 149.0604 [M-H-Glc-CO_2_-H_2_O]^-^ | Geniposidic acid^#^ | RR |
| P9 | 28.424 | C_16_H_24_O_10_ | 421.1461* | 421.1461 [M+HCOOH-H]^-^, 411.1164 [M+Cl]^-^, 375.1389 [M-H]^-^, 213.0785 [M-Glc-H]^-^, 195.0690 [M-H-Glc-H_2_O]^-^, 183.0680 [M-H-Glc-HCHO]^-^ | 6-*O*-methylcatalpol^#^ | SR |
| P10 | 30.338 | C_20_H_30_O_12_ | 461.1781 | 315.1051 [M-H-Rha]^-^, 135.0454 [M-H-Rha-Glc-H_2_O]^-^ | Decaffeoylacteoside | SR, RR |
| P11 | 31.245 | C_16_H_24_O_10_ | 375.1395 | 213.0758 [M-H-Glc]^-^, 169.0878 [M-H-Glc-CO_2_]^-^, 151.0764 [M-H-Glc-CO_2_-H_2_O]^-^ | 8-epiloganic acid | RR |
| P12 | 35.476 | C_21_H_28_O_13_ | 487.1581 | 307.0263 [M-H-Caffeic acid]^-^, 179.0344 [Caffeic acid-H]^-^, 161.0236 [Caffeic acid-H-H_2_O]^-^, 135.0436 [Caffeic acid-H-CO_2_]^-^ | Cistanoside F^#^ | SR, RR |
| P13 | 39.909 | C_21_H_28_O_14_ | 503.1536 | 341.0885 [M-H-Fru]^-^, 323.0754 [M-H-Fru-H_2_O]^-^, 281.0530 [0.2B]^-^, 251.0530 [0.3B]^-^, 221.0456 [0.4B]^-^, 179.0340 [Caffeic acid-H]^-^, 161.0233 [Caffeic acid-H-H_2_O]^-^ | 6-*O*-caffeoyl-α-D-fructofuranosyl-(2→1)-α-D-glucopyranoside | SR |
| P14 | 40.188 | C_14_H_18_O_8_ | 313.0988 | 269.1033 [M-H-CO_2_]^-^, 167.0344 [M-H-Rha]^-^, 123.0447 [M-H-Rha-CO_2_]^-^, 152.0113 [M-H-Rha-CH_3_]^-^ | Rhamnopyranosyl vanilloyl^#^ | RR |
| P15 | 41.470 | C_21_H_32_O_12_ | 475.1940 | 329.1227 [M-H-Rha]^-^, 311.1140 [M-H-Rha-H_2_O]^-^, 149.0594 [M-H-Rha-Glc-H_2_O]^-^ | Darendoside B | SR, RR |
| P16 | 42.881 | C_26_H_40_O_16_ | 607.2434 | 461.1630 [M-H-Rha]^-^, 149.045 [Arabinose-H]^-^, 131.0369 [Arabinose-H-H_2_O]^-^ | β-(3-hydroxy-4-methoxyhenyl) ethyl-*O*-α-L-arabinopyranosyl-(1→6)-*O*-[6-α-L-rhamnpy-ranosyl-(1→3)]-β-D-glucopyranoside | SR |
| P17 | 43.737 | C_15_H_20_O_9_ | 343.1125 | 343.1125 [M-H]^-^, 299.1170 [M-H-CO_2_]^-^, 197.0470 [M-H-Rha]^-^, 182.0232 [M-H-Rha-CH_3_]^-^, 153.0586 [M-H-Rha-CO_2_]^-^, 138.0329 [M-H-Rha-CO_2_-CH_3_]^-^ | Syringic acid-4-*O*-α-L-rhamnoside^#^ |  |
| P18 | 45.248 | C_21_H_28_O_13_ | 487.1596 | 325.0913 [M-H-Fru]^-^, 307.0815 [M-H-Fru-H_2_O]^-^, 163.0398 [Coumaric acid-H]^-^, 145.0294 [Coumaric acid-H-H_2_O]^-^, 341.1052 [M-H-Coumaroyl]^-^ | Acreteoside | SR |
| P19 | 48.170 | C_22_H_30_O_14_ | 517.1719 | 355.1026 [M-H-Fru]^-^, 337.0922 [M-H-Fru-H_2_O]^-^, 295.0827 [0.2B]^-^, 265.0715 [0.3B]^-^, 235.0619 [0.4B]^-^, 193.0503 [Ferulic acid-H]^-^, 175.0395 [Ferulic acid-H-H_2_O]^-^ | 6-*O*-feruloyl-α-D-fructofuranosyl-(2→1)-α-D-glucopyranoside | SR |
| P20 | 49.026 | C_10_H_16_O_3_ | 183.1049 | 183.1016 [M-H]^-^, 139.1138 [M-H-CO_2_]^-^ | Rehmapicrogenin | RR |
| P21 | 49.329 | C_19_H_34_O_8_ | 435.2341* | 389.2164 [M-H]^-^, 435.2172 [M+HCOOH-H]^-^, 161.0390 [Glu-H-H_2_O]^-^ | Rehmaionoside A /Rehmaionoside B | RR |
| P22 | 50.588 | C_35_H_46_O_20_ | 785.2749 | 785.2481 [M-H]^-^, 623.2124 [M-H-Feruloyl]^-^, 461.1504 [M-H-Rha]^-^ | Echinacoside^#^ | RR |
| P23 | 54.366 | C_19_H_34_O_8_ | 435.2356* | 389.2164 [M-H]^-^, 435.2172 [M+HCOOH-H]^-^, 179.0592 [Glu-H]^-^, 161.0473 [Glu-H-H_2_O]^-^ | Rehmaionoside A /Rehmaionoside B | RR |
| P24 | 55.172 | C_9_H_8_O_3_ | 163.0402 | 163.0334 [M-H]^-^, 119.0548 [M-H-CO_2_]^-^ | *p*-Coumaric acid ^a^ | SR |
| P25 | 55.273 | C_24_H_30_O_13_ | 525.1766 | 525.1766 [M-H]^-^, 323.0852 [Caffeoyl+Glc-H]^-^, 201.0796 [M-Caffeoyl-Glc-H]^-^, 183.0716 [M-Caffeoyl-Glc-H_2_O-H]^-^, 179.0365 [Caffeic acid-H]^-^, 161.0252 [Caffeic acid-H_2_O-H]^-^, 135.0452 [Caffeic acid-CO_2_-H]^-^ | 6-*O*-caffeoylharpagide | SR |
| P26 | 55.575 | C_36_H_48_O_20_ | 799.2930 | 799.2632 [M-H]^-^, 623.2171 [M-H-Feruloyl]^-^, 477.1586 [M-H-Rha]^-^, 193.0486 [Ferulic acid-H]^-^ | Jionoside A1/A2 | RR |
| P27 | 57.489 | C_36_H_48_O_20_ | 799.2918 | 799.2632 [M-H]^-^, 623.2171 [M-H-Feruloyl]^-^, 477.1586 [M-H-Rha]^-^, 193.0486 [Ferulic acid-H]^-^ | Jionoside A1/A2 | RR |
| P28 | 58.799 | C_21_H_28_O_12_ | 517.1706* | 323.0963 [M-H-Cinnamic acid]^-^, 219.0642 [0.2B]^-^, 189.0550 [0.3B]^-^, 161.0586 [0.4B]^-^, 147.0449 [Cinnamic acid-H]^-^ | Sibirioside A | SR |
| P29 | 59.202 | C_10_H_10_O_4_ | 193.0510 | 193.0436 [M-H]^-^, 178.0317 [M-CH_3_-H]^-^, 134.0372 [M-H-CO_2_-CH_3_]^-^ | Ferulic acid ^a^ | SR, RR |
| P30 | 61.015 | C_24_H_30_O_12_ | 509.1818 | 509.1818 [M-H]^-^, 545.1595 [M+Cl]^-^, 307.0881 [Coumaroyl+Glc-H]^-^, 201.0783 [M-Coumaroyl-Glc-H]^-^, 163.0407 [Coumaric acid-H]^-^, 145.0303 [Coumaric acid-H-H_2_O]^-^ | 6-*O*-(*p*-coumaroyl) harpagide | SR |
| P31 | 61.116 | C_29_H_36_O_15_ | 623.2152 | 461.1653 [M-H-Caffeoyl]^-^, 315.1106 [M-H-Caffeoyl-Rha]^-^, 179.0348 [Caffeic acid-H]^-^, 161.0241 [Caffeic acid-H-H_2_O]^-^, 135.0441 [Caffeic acid-H-CO_2_]^-^ | Acteoside ^a^ | SR, RR |
| P32 | 62.224 | C_37_H_50_O_20_ | 813.3092 | 813.2784 [M-H]^-^, 637.2302 [M-H-Feruloyl]^-^, 175.0397 [Ferulic acid-H_2_O-H]^-^ | Jionoside B1/jionoside B2 | RR |
| P33 | 62.879 | C_25_H_32_O_13_ | 539.1928 | 539.1928 [M-H]^-^, 337.1015 [Feruloyl+Glc-H]^-^, 193.053 [Ferulic acid-H]^-^, 175.0418 [Ferulic acid-H_2_O-H]^-^ | 6’-*O*-feruloylharpagide | SR |
| P34 | 62.980 | C_35_H_46_O_19_ | 769.2808 | 769.2425 [M-H]^-^, 593.2264 [M-H-Feruloyl]^-^, 447.1623 [M-H-Feruloyl-Rha]^-^, 315.1215 [M-H-Feruloyl-Rha-Ara]^-^, 175.0414 [Ferulic acid-H_2_O-H]^-^ | Scrophuloside B1 / B2^#^ | SR |
| P35 | 63.232 | C_23_H_30_O_12_ | 497.1799 | 497.1799 [M-H]^-^, 533.1621 [M+Cl]^-^, 179.0558 [Glu-H]^-^ | 6-*O*-vanilloylajugol^#^ | SR |
| P36 | 64.843 | C_29_H_36_O_15_ | 623.2186 | 623.1954 [M-H]^-^, 461.1633 [M-H-caffeoyl]^-^, 315.1032 [M-H-Caffeoyl-Rha]^-^, 161.0250 [Caffeic-H-H_2_O]^-^ | Isoacteoside or forsythoside A ^a^ | SR, RR |
| P37 | 65.246 | C_24_H_30_O_13_ | 525.1767 | 345.1247 [M-H-Caffeic acid]^-^, 135.0443 [Caffeic acid-H-CO_2_]^-^, 179.0343 [Caffeic acid-H]^-^, 161.0242 [Caffeic acid-H-H_2_O]^-^ | 8-*O*-caffeoylharpagide^#^ | SR |
| P38 | 67.513 | C_30_H_40_O_16_ | 701.2512* | 701.2169 [M+HCOOH-H]^-^, 655.2457 [M-H]^-^, 507.1867 [M-H-Cinnamic acid]^-^, 147.0457 [Cinnamic acid-H]^-^ | 6-*O*-α-D-galactopyranmosyl harpagoside | SR |
| P39 | 67.715 | C_21_H_34_O_9_ | 429.2252 | 429.2252 [M-H]^-^, 475.2315 [M+HCOOH-H]^-^,465.2035 [M+Cl]^-^ | Jiocarotenoside A1/A2^#^ | RR |
| P40 | 67.765 | C_30_H_38_O_15_ | 637.2345 | 637.2345 [M-H]^-^, 461.1797 [M-H-Feruloyl]^-^, 315.1143 [M-H-Feruloyl-Rha]^-^, 193.0532 [Ferulic acid-H]^-^, 175.0423 [Ferulic acid-H-H_2_O]^-^ | Leucosceptoside A | RR |
| P41 | 69.327 | C_36_H_48_O_19_ | 783.2996 | 607.2139 [M-H-Feruloyl]^-^, 193.0564 [Ferulic acid-H]^-^, 175.0381 [Ferulic acid-H-H_2_O]^-^ | Angoroside C | SR, RR |
| P42 | 70.133 | C_36_H_48_O_19_ | 783.2961 | 607.2268 [M-H-Feruloyl]^-^, 461.1671 [M-H-Feruloyl-Rha]^-^, 193.0514 [Ferulic acid-H]^-^, 175.0404 [Ferulic acid-H-H_2_O]^-^ | Isoangoroside C | SR, RR |
| P43 | 70.233 | C_24_H_30_O_12_ | 509.1808 | 345.1132 [M-H-Coumaric acid]^-^, 201.0732 [M-H-Coumaroyl-Glc]^-^, 183.0678 [M-H-Coumaric acid-Glc]^-^, 163.0399 [Coumaric acid-H]^-^, 145.0292 [Coumaric acid-H-H_2_O]^-^ | 8-*O*-(*p*-coumaroyl) harpagide | SR |
| P44 | 70.687 | C_30_H_40_O_16_ | 701.2519* | 507.1708 [M-H-Cinnamic acid]^-^, 323.1020 [Glc+Glc-H]^-^ or [Glc+Gal-H]^-^, 183.0661 [M-H-disaccharide]^-^, 147.0440 [Cinnamic acid-H]^-^ | 6"-*O*-α-D-glucopyranosylharpagoside or 6"-*O*-α-D-galactopyranosylharpagoside or another isomer | SR |
| P45 | 70.838 | C_18_H_22_O_9_ | 381.1285 | 381.1285 [M-H]^-^, 193.0512 [Ferulic acid-H]^-^, 175.0416 [Ferulic acid-H-H_2_O]^-^ | Ningposide A /B | SR |
| P46 | 71.593 | C_25_H_32_O_13_ | 539.1924 | 345.1244 [M-H-Ferulic acid]^-^, 183.0653 [M-H-Ferulic acid-Glc]^-^, 193.0507 [Ferulic acid-H]^-^, 175.0390 [Ferulic acid-H-H_2_O]^-^ | 8-*O*-feruloylharpagide^#^ | SR |
| P47 | 72.047 | C_30_H_38_O_15_ | 637.2331 | 637.2331 [M-H]^-^, 461.1797 [M-H-Feruloyl]^-^, 315.1190 [M-H-Feruloyl-Rha]^-^, 193.0501 [Ferulic acid-H]^-^, 161.0248 [Glu-H_2_O-H]^-^ | Leucosceptoside A or isomer | RR |
| P48 | 72.450 | C_30_H_38_O_14_ | 621.2144 | 621.2144 [M-H]^-^, 459.1544 [M-H-Glc]^-^ | Nigroside I/II^#^ | RR |
| P49 | 73.256 | C_21_H_36_O_10_ | 493.2286* | 447.2212 [M-H]^-^, 315.1803 [M-H-Api]^-^ | Borneol 7-*O*-[β-D-apiofuranosyl-(1→6)]-β-D-glucopyranoside^#^ | OR |
| P50 | 74.011 | C_24_H_30_O_12_ | 509.1827 | 509.1827 [M-H]^-^, 345.1239 [M-H-2-hydroxycinnamoyl-H_2_O]^-^, 201.0805 [M-H-2-hydroxycinnamoyl-Glc]^-^, 163.0412 [2-hydroxycinnamic acid-H]^-^ | 8-*O*-(2-hydroxycinnamoyl) harpagide^#^ | SR |
| P51 | 74.918 | C_24_H_30_O_11_ | 539.1759* | 539.1769 [M+HCOOH-H]^-^, 345.1175 [M-H-Cinnamic acid]^-^, 183.0655 [M-H-Cinnamic acid-Glc]^-^, 147.0450 [Cinnamic acid-H]^-^ | 6''-O-cinnamoylharpagide^#^ | SR |
| P52 | 75.220 | C_31_H_40_O_15_ | 651.2496 | 475.1757 [M-H-Feruloyl]^-^, 329.1066 [M-H-Feruloyl-Rha]^-^, 193.0500 [Ferulic acid-H]^-^, 175.0395 [Ferulic acid-H-H_2_O]^-^ | Cistanoside D | SR, RR |
| P53 | 77.286 | C_38_H_50_O_20_ | 825.3091 | 825.2774 [M-H]^-^, 783.2624 [M-H-acetyl]^-^, 607.2160 [M-H-Feruloyl-acetyl]^-^, 443.1582 [M-H-Feruloyl-acetyl-Rhamnosyl-H_2_O]^-^, 193.0524 [Ferulic acid-H]^-^, 175.0395 [Ferulic acid-H-H_2_O]^-^ | Acetylangoroside C^#^ | SR |
| P54 | 78.646 | C_31_H_40_O_15_ | 651.2291 | 505.1713 [M-H-Rha]^-^, 475.1797 [M-H-feruloyl]^-^, 329.1223 [M-H-Feruloyl-Rha]^-^, 193.0502 [Ferulic acid-H]^-^ | Martynoside | SR, RR |
| P55 | 79.325 | C_24_H_30_O_11_ | 539.1759* | 987.3460 [2M-H]^-^, 539.1734 [M+HCOOH-H]^-^, 345.1179 [M-H-Cinnamic acid]^-^, 146.9661 [Cinnamic acid-H]^-^, 183.1190 [M-H-Cinnamic acid-Glc]^-^ | Harpagoside ^a^ | SR |
| P56 | 80.131 | C_9_H_8_O_2_ | 147.0461 | 147.0389 [M-H]^-^, 103.0554 [M-H-CO_2_]^-^ | Cinnamic acid ^a^ | SR |
| P57 | 82.766 | C_28_H_40_O_10_ | 535.2715 | 535.2706 [M-H]^-^, 163.0390 [Coumaric acid-H]^-^, 145.0306 [Coumaric acid-H_2_O-H]^-^ | Frehmaglutoside C or isomer ^#^ | RR |
| P58 | 85.093 | C_28_H_40_O_10_ | 535.2704 | 535.2716 [M-H]^-^, 163.0403 [Coumaric acid-H]^-^, 145.0303 [Coumaric acid-H_2_O-H]^-^ | Frehmaglutoside C or isomer | RR |
| P59 | 92.700 | C_39_H_62_O_15_ | 815.4347* | 815.4347 [M+HCOOH-H]^-^, 769.4271 [M-H]^-^, 805.4058 [M+Cl]^-^ | Ophiopogonin R^#^ | OR |
| P60 | 94.060 | C_44_H_70_O_18_ | 931.4864* | 885.4456 [M-H]^-^, 753.4044 [M-H-Xyl]^-^, 607.3460 [M-H-Xyl]^-^ | Ophiopojaponin C^#^ | OR |
| P61 | 94.866 | C_39_H_62_O_14_ | 799.4397* | 753.4057 [M-H]^-^, 607.3468 [M-H-Rha]^-^ | Ophiopogonin Ra^#^ | OR |
| P62 | 95.873 | C_18_H_18_O_7_ | 345.1066 | 345.1066 [M-H]^-^, 330.2448 [M-CH_3_-H]^-^, 209.0489 [M-H-*B-ring*-2CH_2_]^-^ | 5,7,2',4'-tetradihydroxy-8-methoyl-6-methyl-homoisoflavanone^#^ | OR |
| P63 | 97.485 | C_44_H_70_O_17_ | 915.4897* | 869.4521 [M-H]^-^，737.416 [M-H-Xyl]^-^ | (25R) -alphas-3β, 14'-dihydroxy-3-*O*-acenaphthopyranosyl- (1 → 2) - [β-D-xylopyranosyl] -β-D - glucopyranoside^#^ | OR |
| P64 | 97.838 | C_20_H_22_O_7_ | 373.1383 | 373.1296 [M-H]^-^, 343.0797 [M-H-2CH_3_]^-^, 222.0536 [M-H-*B-ring*-CH_2_]^-^, 207.0312 [M-H-*B-ring*-CH_2_-CH_3_]^-^ | 5,7,2'-trihydroxy-3',5'-dimethoxy-6,8-dimethyl-homoisoflavanone^#^ | OR |
| P65 | 98.442 | C_41_H_64_O_15_ | 841.4510* | 841.4228 [M+HCOOH-H]^-^, 795.4168 [M-H]^-^, 753.4038 [M-C_2_H_2_O-H]^-^, 735.3921 [M-C_2_H_2_O-H_2_O-H]^-^, 607.3510 [M-C_2_H_2_O-Rha-H]^-^, 445.2954 [M-C_2_H_2_O-Rha-Glc-H]^-^ | Ophiogenin 2-*O*-Acetyl-α-L- rhamnopyranosyl-(1→2)-β-D-glucopyranoside^#^ | OR |
| P66 | 98.644 | C_39_H_62_O_13_ | 783.4441* | 783.4034 [M+HCOOH-H]^-^, 737.4358 [M-H]^-^, 591.3695 [M-Rha-H]^-^, 163.1695 [Rhamnose-H]^-^, 145.0541 [Rha-H]^-^ | prazarigenin A 3-*O*-α–L–rhamnopyranosyl– (1–2)–β–D–glucopyranoside | OR |
| P67 | 99.752 | C_41_H_64_O_15_ | 841.4509* | 841.4509 [M+HCOOH-H]^-^, 831.4213 [M+Cl]^-^, 445.1283 [M-C_2_H_2_O-Rha-Glc-H]^-^ | Ophiogenin 4-*O*-Acetyl-α-L- rhamnopyranosyl-(1→2)-β-D-glucopyranoside^#^ | OR |
| P68 | 100.004 | C_17_H_16_O_5_ | 299.0983 | 299.0983 [M-H]^-^, 165.0205 [M-H-*B-ring*+H-CH_2_-CO]^-^ | 5,7-dihydroxy-6-methy-3-(4'-hydroxybenzyl) chromone-4-one^#^ | OR |
| P69 | 100.961 | C_46_H_72_O_18_ | 957.5046* | 957.4712 [M+COOH-H]^-^, 591.3567 [M-C_2_H_2_O-Xyl-Rha-H]^-^, 737.4048 [M-C_2_H_2_O-Xyl-H]^-^, 869.4532 [M-C_2_H_2_O-H]^-^ | Prazarifenin A 3-O-[2-O-Acetyl-α-L-rhamnopyranosyl-(1→2)-[β-D-xylopyranosyl-(1→4)]-β-D-glucopyranoside] ^#^ | OR |
| P70 | 101.918 | C_20_H_22_O_7_ | 373.1392 | 373.1392 [M-H]^-^, 429.0946 [M+Cl]^-^, 207.1418 [M-H-*B-ring*-CH_2_+H]^-^ | 5,7,4'-trihydroxy-3',5'-dimethoxy-6,8-dimethyl hamoisoflavanone^#^ | OR |
| P71 | 102.673 | C_41_H_64_O_14_ | 825.4556* | 825.4556 [M+HCOOH-H]^-^, 779.4471 [M-H]^-^, 719.4228 [M-C_2_H_2_O-H_2_O-H]^-^, 591.3665 [M-C_2_H_2_O-Rha-H]^-^ | Prazarifenin A 3-*O*-[2-O-Acetyl-α-L-rhamnopyranosyl-(1→2)-β-D-glucopyranoside] ^#^ | OR |
| P72 | 103.127 | C_19_H_20_O_6_ | 343.1267 | 343.1172 [M-H]^-^, 207.0655 [M-H-*B-ring*-CH_2_+H]^-^, 179.0685 [M-H-*B-ring*+H-CH_2_-CO]^-^, 149.0187 [M-H-*B-ring*+H-CH_2_-CO-HCHO]^-^ | 5,7,3'-trihydroxy-4'-methoxy-6,8-dimethyl hamoisoflavanone or 5,7,2'-trihydroxy-4'-methoxy-6,8-dimethyl hamoisoflavanone | OR |
| P73 | 103.127 | C_44_H_70_O_16_ | 899.4967^*^ | 853.4562 [M-H]^-^, 721.4149 [M-H-Xyl]^-^, 575.3639 [M-H-Xyl-Ara]^-^ | Ophiopogonin D | OR |
| P74 | 103.479 | C_19_H_20_O_7_ | 359.1236 | 359.1126 [M-H]^-^, 344.0885 [M-H-CH_3_]^-^, 223.1226 [M-H-*B-ring*-CH_2_+H]^-^, 208.0974 [M-H-*B-ring*-CH_2_+H-CH_3_]^-^ | Ophiopogonanone E | OR |
| P75 | 105.293 | C_19_H_20_O_6_ | 343.1269 | 343.1180 [M-H]^-^, 207.0645 [M-H-*B-ring*-CH_2_+H]^-^ | 5,7,4'-trihydroxy-5'-methoxy-6,8-dimethyl hamoisoflavanone | OR |
| P76 | 105.847 | C_19_H_16_O_7_ | 355.0915 | 401.0984 [M+HCOOH-H]^-^, 355.0915 [M-H]^-^ | 5,7-dihydroxy-6,8-dimethyl-3-(2'-hydroxy-3',4'-methylenedioxybenzyl) chromone^#^ | OR |
| P77 | 107.257 | C_18_H_16_O_6_ | 327.0956 | 327.0956 [M-H]^-^, 192.0422 [M-H-*B-ring*-CH_2_]^-^ | Ophiopogonanone A | OR |
| P78 | 109.738 | C_19_H_16_O_6_ | 339.0956 | 339.0956 [M-H]^-^, 311.2349 [M-H-CO]^‑^ | Methylophiopogone A | OR |
| P79 | 110.179 | C_19_H_18_O_6_ | 341.1137 | 341.1137 [M-H]^-^, 206.0581 [M-H-*B-ring*-CH_2_]^-^, 178.0629 [M-H-*B-ring*-CH_2_-CO]^-^ | Methylophiopogonanone A | OR |
| P80 | 110.985 | C_19_H_20_O_5_ | 327.1325 | 327.1325 [M-H]^-^, 206.0586 [M-H-*B-ring*-CH_2_]^-^, 178.0628 [M-H-*B-ring*-CH_2_-CO]^-^ | Methylophiopogonanone B | OR |

Note: t_R_, retention time; *, [M+HCOOH-H]^-^; Glc, glucosyl group, C_6_H_10_O_5_, 162Da; Glu, glucose, C_6_H_12_O_6_, 180Da; Rha, rhamnosyl group, C_6_H_10_O_4_,146Da; Fru, fructosyl group, C_6_H_10_O_5_, 162Da; Xyl, xylosyl group, C_5_H_8_O_4_, 132Da. ^#^, additional compounds were detected compared to the previous ZYD study. ^a^, Identiﬁed by comparing with reference standards. SR, *Scrophulariae Radix*; RR, *Rehmanniae Radix*; OR, *Ophiopogonis Radix*.
